# Intramedullary Nailing of Intertrochanteric Femoral Fractures in a Level I Trauma Center in Finland: What Complications Can be Expected?

**DOI:** 10.1097/CORR.0000000000002792

**Published:** 2023-08-15

**Authors:** Miika Lähdesmäki, Antti AJ Ylitalo, Leevi Karjalainen, Mikko Uimonen, Ville M. Mattila, Jussi P. Repo

**Affiliations:** 1 Faculty of Medicine and Health Technology, Tampere University, Tampere, Finland; 2 Department of Orthopaedics and Traumatology, Tampere University Hospital, Tampere, Finland; 3 Department of Surgery, Hospital Nova of Central Finland, Jyväskylä, Finland

## Abstract

**Background:**

Intertrochanteric hip fractures are one of the most common fractures in older people, and the number is estimated to increase. These fractures are often treated with intramedullary nailing; however, various complications have been reported. It is important to identify the potential complications and investigate whether the choice of implant and patient-related factors are associated with the risk of complications to develop better strategies for preventing them.

**Questions/purposes:**

(1) In the treatment of intertrochanteric fractures with intramedullary nailing, what are the risks of major complications and 30-day mortality? (2) Which implant types are associated with greater odds of major complications? (3) Which patient-related factors are associated with increased odds of major complications?

**Methods:**

In this retrospective, comparative study, we reviewed the health records of 2397 patients with a femoral fracture treated at one Level I trauma center between January 2014 and November 2020. Of those, we considered patients who were treated with intramedullary nailing for an intertrochanteric fracture after sustaining a low-energy injury as potentially eligible. Based on this criterion, 53% (1279) were eligible; a further 47% (1118) were excluded because the fixation method was other than intramedullary nailing, the fracture pattern was other than intertrochanteric fracture, or the fracture was caused by a high-energy injury mechanism. Another 4% (97) were excluded because they had incomplete datasets because of follow-up less than 12 months, leaving 49% (1182) for analysis. During the study period, intramedullary nails were generally used to treat nearly all intertrochanteric fractures at our hospital. The risk of complications was then assessed by chart review. Acute myocardial ischemia, cutout, nail breakage, pulmonary embolism, sepsis, stroke, and wound infection were defined as major complications. Cutout, nail breakage, and wound infection were defined as major complications leading to reoperation. To examine the association of implant type and major complications, a logistic regression analysis was performed. Additionally, the risks of major complications leading to reoperation were compared between implants. Finally, a univariable logistic regression analysis was performed to examine the association between patient-related factors and major complications.

**Results:**

The overall proportion of patients experiencing complications was 16% (183 of 1182), and the crude percentage of 30-day mortality was 9% (107 of 1182) based on the hospital`s medical records. After controlling for patient-related factors such as disease, age, and smoking, we found that nail type was not associated with odds of major complications leading to reoperation (Gamma3: OR 0.86 [95% CI 0.44 to 1.67]; p = 0.67; Trochanteric Fixation Nail: OR 0.61 [95% CI 0.2 to 1.53]; p = 0.33; Proximal Femoral Nail Antirotation: OR 0.55 [95% CI 0.16 to 1.49]; p = 0.29) compared with the Trochanteric Fixation Nail Advanced. Anticoagulation (OR 1.70 [95% CI 1.11 to 2.59]; p = 0.01), congestive heart failure (OR 1.91 [95% CI 1.13 to 3.11]; p = 0.01), and hypertension (OR 1.67 [95% CI 1.08 to 2.63]; p = 0.02) were associated with a major complication. Liver disease (OR 5.19 [95% CI 0.78 to 20.8]; p = 0.04) was associated with a major complication leading to reoperation.

**Conclusion:**

This study provides a better understanding of the occurrence of surgical and medical complications after intramedullary nailing of intertrochanteric fractures. The new-generation nail types are comparable options based on the risk of reoperation. Anticoagulation, congestive heart failure, and hypertension were associated with major complications, highlighting the need for careful management and monitoring of these comorbidities during intramedullary nailing procedures.

*Level of Evidence* Level III, therapeutic study.

## Introduction

Intertrochanteric hip fractures are among the most common fractures in older people and are known to be associated with high morbidity [6, 12, 15]. The advantages of intramedullary nailing for the patient are early mobilization and weightbearing. However, various complications have been reported after intramedullary nailing of intertrochanteric fractures [20]. The complications associated with intramedullary nailing can be divided into medical complications, such as cardiovascular complications, and surgical complications, which include surgical site infections and mechanical complications. Because the described population consists of mostly frail elderly people, the most observed complications are nonsurgical such as pneumonia [2, 18, 27, 28]. The most frequent mechanical complication is cutout, with the risk varying between 1% and 5% [2, 3, 25, 30]. Two studies reported higher cutout rates when a nail type with a helical blade was used than for a nail with femoral neck screw [4, 32], whereas another study found no difference [11]. Another clinically important mechanical complication is nail breakage, which can occur in 0.2% to 0.88% of patients and can result in severe disability [8, 25, 30]. A few studies have evaluated the risk of nail breakage with the Trochanteric Fixation Nail Advanced (TFNA) compared with that of other nail types [11, 30, 35] after an implant retrieval study of TFNA nails reported a unique fracture pattern with a stepped propagation pathway [16]. The risk of surgical site infections, which can prolong hospitalization, is reported to be 1.5% to 2.8% [2, 12]. Factors such as male sex, diabetes, and long delays between fracture and operation are associated with higher morbidity and mortality [1]. In another study, only a high Charlson comorbidity index (≥ 3) was identified as a risk factor for a complication, whereas delay of surgery was not a risk factor [9]. Hence, further research is required, because there have been contradictory results [9, 10].

There are many studies investigating specific complications associated with nailing of intertrochanteric fractures. Our goal was to provide a better understanding of the occurrence of surgical and medical complications, as well as complications associated with intramedullary nailing of intertrochanteric fractures. In addition, it is unclear whether the risk of reoperation owing to complications such as implant breakage, cutout, and wound infection is equivalent between new-generation intramedullary nail types.

We therefore asked, (1) In the treatment of intertrochanteric fractures with intramedullary nailing, what are the risks of major complications and 30-day mortality? (2) Which implant types are associated with greater odds of major complications? (3) Which patient-related factors are associated with increased odds of major complications?

## Patients and Methods

### Study Design and Setting

A retrospective, comparative study was conducted using the patient record database of Tampere University Hospital, Tampere, Finland. Tampere University Hospital is a tertiary-level hospital serving as a trauma care unit for the surrounding three hospital districts. Tampere University Hospital has a catchment area population of approximately 900,000 inhabitants. After defining the criteria of complications, data were collected by two of the authors (ML and LK) by reviewing individual patients’ health records and radiologic images. Institutional permission was granted by the head of the department.

### Patients

Patient information was obtained using the Nordic Medico-Statistical Committee procedure codes NFB10 (primary partial prosthetic replacement of the hip not using cement), NFB30 (primary total prosthetic replacement of the hip not using cement), NFB60 (demanding prosthetic replacement of the hip), NFB99 (other primary prosthetic replacement of the hip), NFJ54 (internal fixation of fracture of the upper femur with intramedullary nailing), NFJ62 (internal fixation of fracture of other parts of the femur with plate), NFJ64 (other internal fixation of fracture of other parts of the femur), NFJ84 (refixation of fracture of the femur), NFJ86 (late operation of fracture of the femur to promote bone formation), NFJ99 (other fracture surgery of the femur), NFU20 (removal of an internal fixation device from the femur), and NFU99 (removal of other implant from the hip or femur).

**Fig. 1:**
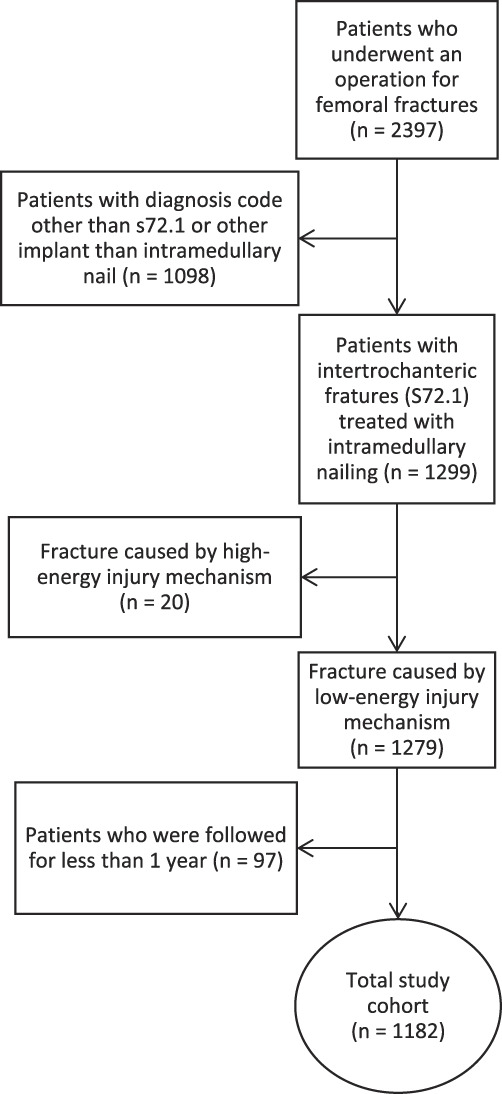
This flowchart represents patient selection.

We reviewed the health records of 2397 patients with a femoral fracture who underwent surgery between January 2014 and November 2020. We considered patients who were treated with intramedullary nailing for an intertrochanteric fracture (International Classification of Diseases, Tenth Revision, diagnosis code S72.1) after sustaining a low-energy injury as potentially eligible. Based on that criterion, 53% (1279) were eligible; a further 47% (1118) were excluded because fixation was other than intramedullary nailing, the fracture pattern was other than an intertrochanteric fracture, or the fracture was caused by a high-energy injury mechanism. Another 4% (97) were excluded because they had incomplete datasets because of follow-up less than 12 months, leaving 49% (1182) for analysis (Fig. 1). The mean follow-up of the study population was 4 years (range 12 months to 7.5 years). During the study period, intramedullary nails were used to treat nearly all intertrochanteric fractures.

### Descriptive Data

The mean age of the patients was 82 ± 10 years at the time of the operation, and 73% of the patients (865 of 1182) were female. The mean delay from fracture to surgery was 2 ± 2 days. (Table 1).

**Table 1.** Sociodemographic and clinical details (n = 1182)

Characteristic	Value
Age in years	82 (24 to 104)
Female patients	73% (865)
ASA class^a^	3 (1 to 4)
1	0.3% (4)
2	9% (110)
3	66% (776)
4	24% (288)
Previous hip fracture	14% (170)
Renailing^b^	1% (9)
CCI score^c^	6 (0 to 16)
Myocardial infarction	5% (57)
Congestive heart failure	14% (164)
PVD or CAD	25% (300)
CVA or TIA	16% (184)
Dementia	34% (397)
COPD	6% (76)
Connective tissue disease	8% (93)
Peptic ulcer disease	0.1% (1)
Liver disease	1% (16)
Mild	1% (11)
Moderate or severe	0.4% (5)
Diabetes mellitus	20% (235)
Uncomplicated	19% (224)
Complicated	1% (11)
Hemiplegia	4% (42)
Kidney failure (moderate or severe)	5% (56)
Tumor	17% (205)
Localized	14% (170)
Metastatic	3% (35)
Leukemia	0.2% (2)
Lymphoma	0.4% (5)
AIDS	0% (0)
Osteoporosis	9% (101)
Hypertension	58% (683)
Anticoagulation	29% (339)
Smoking^d^	7% (85)
Trauma under the influence of alcohol^e^	4% (51)
Living at home	70% (832)
Days from trauma to operation^f^	2 (0 to 23)
Exitus within 30 days	9% (107)

Numbers are presented as % (n) or mean (range).

a Classification system of physical status. Data missing for 4 (0.3%) patients.

b Same femur that was operated on previously with intramedullary nailing.

c Predicts 10-year mortality considering comorbidities.

d History of tobacco smoking not recorded.

e Blood alcohol level of at least 0.5 per mille in electronic record.

f Number of complete days between hip fracture and surgery. ASA = American Society of Anesthesiologists; CCI = Charlson comorbidity index; PVD = peripheral vascular disease; CAD = coronary artery disease; CVA = cerebrovascular accident; TIA = transient ischemic attack; COPD = chronic obstructive pulmonary disease.

### Variables, Outcome Measures, Data Sources, and Bias

Collected demographic variables included age; sex; International Classification of Diseases, Tenth Revision diagnosis codes (S72.0, S72.1, S72.2, S72.3, and S72.4); implant information; procedure details; complication type; time to complications and possible comorbidities; and the risk factors for patients (Charlson comorbidity index and risk factors included in the Charlson comorbidity index, American Society of Anesthesiologists grade, use of anticoagulants, smoking, trauma sustained while under the influence of alcohol, and information on previous hip fractures). Trauma was categorized as low-energy or high-energy based on the injury mechanism. A low-energy fracture was defined as a fracture sustained during a fall on the same level, fall from stairs, or fall from bed. A high-energy fracture was determined to be a fracture resulting from a motor vehicle collision, crush accident, or fall from heights (other than a fall from bed or a fall from stairs).

The present study is a single-center study and thus may be affected by selection bias (minimizing generalizability of these results to patients residing in more rural areas, those in a different economic class, or those presenting to hospitals using different implants), transfer bias (because more complications may have occurred if the study been run longer or if patients had lived longer), and bias associated with human data entry and collection.

### Primary and Secondary Study Outcomes

Our primary study goal was to investigate a comprehensive overview of the occurrence of major complications, including surgical and medical complications, that may have a major impact on patients' well-being or may require prolonged hospitalization or surgical procedures. To achieve this, we recorded complications through a chart review and studied the risks.

Our secondary study goals were to investigate whether there was an association between patient-related risk factors and complications and whether the choice of implant has an impact on the risk of undergoing revision surgery. To achieve these, we set the TNFA (Depuy Synthes) as the reference implant and calculated the ORs of other implants against those of the reference implant. Lastly, a univariable logistic regression analysis was performed to examine the association between patient-related factors and major complications.

We recorded the following medical complications: pneumonia, acute myocardial ischemia, sepsis, stroke, pulmonal embolism, and pyelonephritis. A medical complication was recorded if it was clinically diagnosed and treated within 3 weeks postoperatively at Tampere University Hospital.

The following surgical complications were recorded: cutout, nail breakage, peri-implant fracture, nonunion, malunion, femoral neck screw migration without cutout, oversized femoral neck screw, intolerable pain, wound infection, and hematoma. Postoperative peri-implant fracture was defined as a complication that occurred within 2 weeks postoperatively. For all other surgical complications, the surveillance was a minimum of 12 months to comprehensively detect complications such as nail breakages occurring after 1 year.

Of all recorded complications, acute myocardial ischemia, cutout, nail breakage, pulmonary embolism, sepsis, stroke, and wound infection were defined as major complications. For all of the other complications, such as pneumonia and hematoma, we studied only the risks but did not include them in the analysis of associations with major complications. Cutout, nail breakage, and wound infection were defined as major complications leading to reoperation.

Nonunion was defined as no sign of bone healing resulting in reoperation. Malunion was defined as a fracture that healed in an inadequate position, resulting in reoperation.

Cutout was defined as extrusion of the femoral neck screw or helical blade from the femoral head. Femoral neck screw migration was defined as migration of the femoral neck screw or helical blade without extrusion from the femoral head, resulting in reoperation.

Postoperative peri-implant fracture was defined as a secondary fracture around the distal tip of the nail occurring within 2 weeks postoperatively. Iatrogenic peri-implant fracture was defined as a secondary fracture caused perioperatively around the distal tip of the nail.

An oversized femoral neck screw was defined as a complication when it resulted in reoperation because the femoral neck screw was slightly longer than intended, and the patient continued to experience discomfort because of tenderness around the prominent tip of the helical blade, even though the fracture had healed without any issues. As a result, the nail was removed. Wound infections and hematomas were defined as complications when they resulted in revision. Intolerable pain was defined as consistent pain of the hip resulting in reoperation. The decision to reoperate was made when pain in the operated-on hip or thigh was not alleviated, despite the absence of other abnormal radiologic or clinical findings.

### Description of Surgery

Tampere University Hospital also serves as a teaching hospital. In the present study, fractures were treated by senior orthopaedic surgeons or residents in orthopaedics and traumatology at the end of their specialization program who had an average of 6 years of postgraduate education. The choice of the implant type was primarily influenced by the availability of nails through our procurement process at that time. There was rarely more than one implant type available, in which case, the surgeon's preference might have an impact on implant choice. Overall, 42% (492 of 1182) of the fractures were treated with the TFNA, 31% (368 of 1182) were treated with the Gamma3 (Stryker), 15% (174 of 1182) were treated with the Trochanteric Fixation Nail ([TFN] DePuy Synthes), 12% (146 of 1182) were treated with the proximal Femoral Nail Antirotation ([PFNA] DePuy Synthes), and 0.2% (two of 1182) were treated with the Intertan nail (Smith & Nephew). Femoral neck screws were used in 77% (905 of 1182) of operations and helical blades were used in 23% (276 of 1182). Open reduction was performed in 20% (230 of 1182) of the operations when sufficient reduction was not achieved with closed methods. Cerclage wiring was used in 4% of the fractures (42 of 1182) to hold fractured bone fragments.

### Ethical Approval

Ethical committee evaluation was not sought because of the retrospective register-based study design and because patients were not contacted.

### Statistical Analysis

Data on continuous variables are presented as means and SDs when normally distributed and medians and IQRs when non-normally distributed. Data on discrete variables are presented as percentages and frequencies.

Information on major complications was extracted from the patient record database at Tampere University Hospital, including patient files and radiographs. To examine the association of the implant with major complications, a logistic regression analysis was performed with the implant as an independent variable and the occurrence of any major complication as a dependent variable. The following served as adjusting variables in the analysis: congestive heart failure, previous myocardial infarction, peripheral vascular disease, previous stroke, hemiparesis, dementia, diabetes, liver insufficiency, connective tissue disorder, chronic obstructive pulmonary disease, malignancy, renal insufficiency, hypertension, osteoporosis, psychiatric disorder, smoking, use of anticoagulative medication, age, sex, living at home (yes or no), and whether the treated fracture was related to alcohol misuse. TFNA was set as the reference implant, and the ORs of the other implants were then calculated against those of the reference implant.

The risk of major complications leading to reoperation was examined and compared between implants.

Lastly, a univariable logistic regression analysis was performed to examine the association between patient-related factors and major complications. Because the data analysis was simplified, in which the analyzed data contained only one variable, statistical bias was low. The statistical analysis was performed using R (4.0.3, R Foundation for Statistical Computing) statistical software. Statistical significance was defined as p < 0.05.

## Results

### Major Complications and 30-day Mortality

**Fig. 2:**
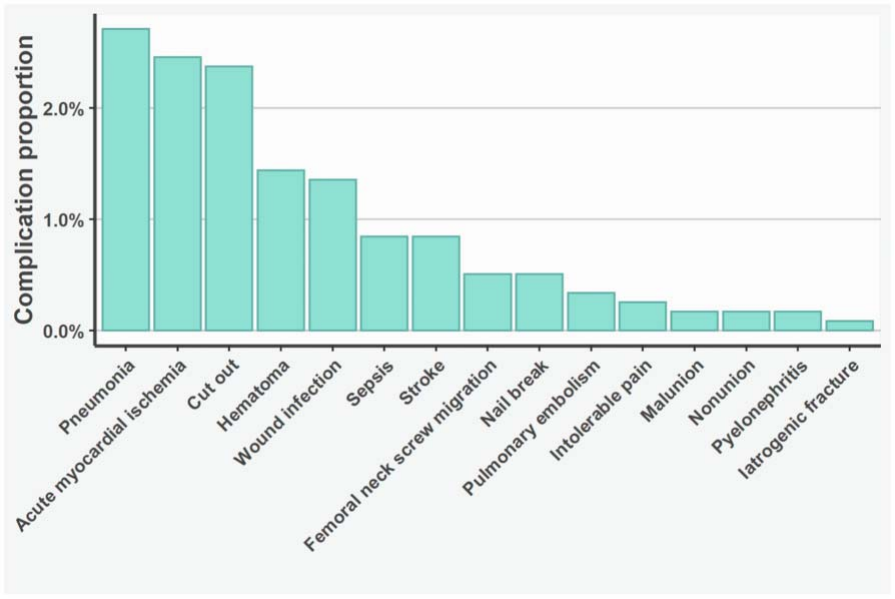
This graph shows the risk of major complications for all 1182 patients.

The overall risk of major complications was 16% (183 of 1182). The median time from the initial operation to the diagnosis of a major complication was 16 days (range 0 to 707 days). In total, 45% (83 of 183) of complications occurred within 2 weeks of the operation and 7% (12 of 183) of complications occurred after 1 year (Fig. 2). The crude percentage of mortality was 9% (107 of 1182) based on information from the patients’ files. Pneumonia occurred in 2.7% (32 of 1182), acute myocardial ischemia in 2.5% (29 of 1182), cutout in 2.4% (28 of 1182), hematoma in 1.4% (17 of 1182), and wound infection in 1.4% (16 of 1182) of patients (Table 2).

**Table 2.** Proportions of patients experiencing complications (n = 1182)

Complication	Value
Medical complications	
Acute myocardial ischemia	2.5% (29)
Pneumonia	2.7% (32)
Pulmonary embolism	0.3% (4)
Pyelonephritis	0.2% (2)
Sepsis	0.8% (10)
Stroke	0.8% (10)
Surgical complications	
Cutout	2.4% (28)
Femoral neck screw migration	0.5% (6)
Hematoma	1.4% (17)
Iatrogenic fracture	0.1% (1)
Intolerable pain	0.3% (3)
Malunion	0.2% (2)
Nail breakage	0.5% (6)
Nonunion	0.2% (2)
Wound infection	1.4% (16)

### Association Between Implant Types and Complications

**Fig. 3:**
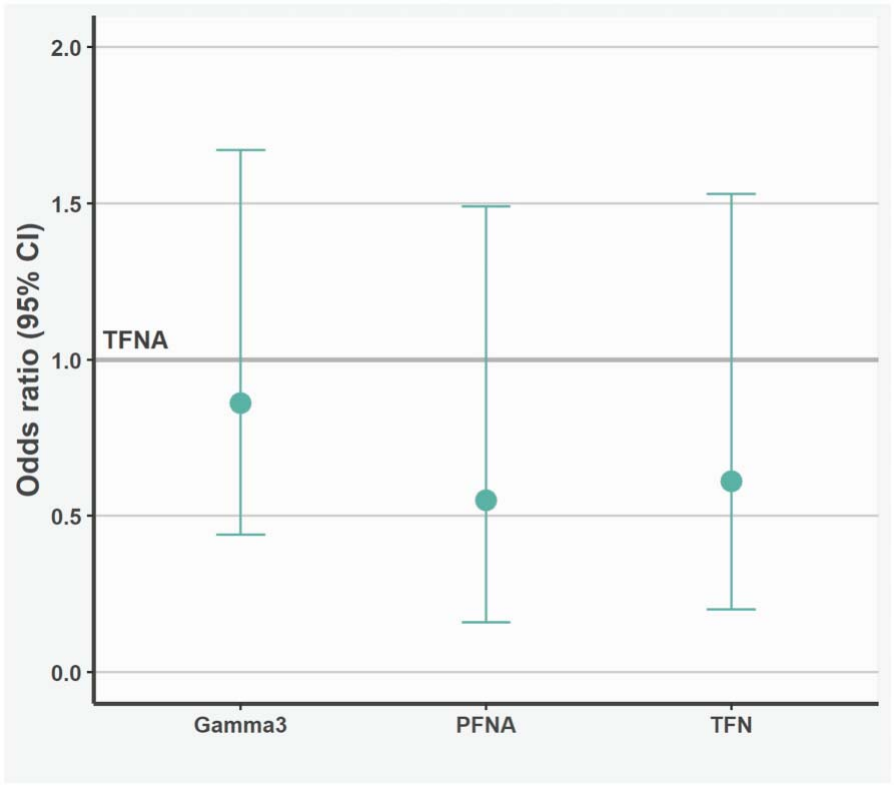
This graph shows the adjusted ORs for major complications for implant types, with the TFNA implant set as a reference. Points represent OR and whiskers show 95% CI.

**Fig. 4:**
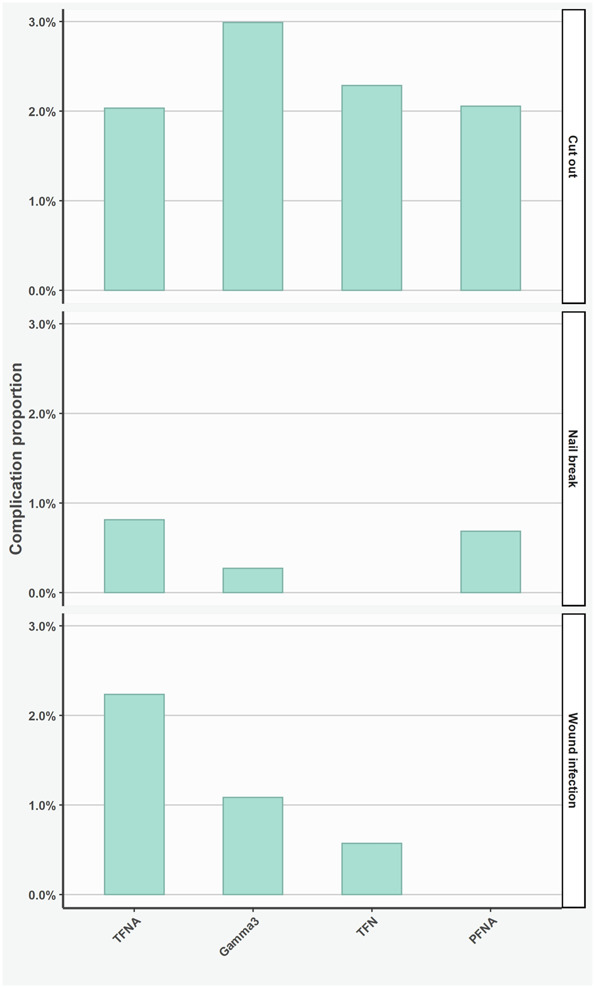
This graph shows major complications of implants leading to reoperation.

Nail type was not associated with the odds of major complications leading to reoperation (Gamma3: OR 0.86 [95% CI 0.44 to 1.67]; p = 0.67; TFN: 0.61 [95% CI 0.2 to 1.53]; p = 0.33; PFNA: OR 0.55 [95% CI 0.16 to 1.49]; p = 0.29) in relation to the TFNA nail (Fig. 3). In the assessment of specific complication types, the risk of cutout was 3.0% (11 of 368) for Gamma3, 2.3% (four of 174) for TFN, 2.1% (three of 146) for PFNA, and 2.0% (10 of 492) for TFNA (Fig. 4). The risk of nail breakage was 0.8% (four of 492) for TFNA, 0.7% (one of 146) for PFNA, and 0.3% (one of 368) for Gamma3 (Table 3). Overall, 3.3% (nine of 276) of nails with helical blades and 2.1% (19 of 905) of nails with femoral neck screws developed cutout.

**Table 3.** Proportions of patients experiencing complications by implant

Complication	Risk
Cutout	
Gamma3	3.0% (11 of 368)
PFNA	2.1% (3 of 146)
TFN	2.3% (4 of 174)
TFNA	2.0% (10 of 492)
Nail breakage	
Gamma3	0.3% (1 of 368)
PFNA	0.7% (1 of 146)
TFN	0% (0 of 174)
TFNA	0.8% (4 of 492)
Wound infection	
Gamma3	1.1% (4 of 368)
PFNA	0% (0 of 146)
TFN	0.6% (1 of 174)
TFNA	2.2% (11 of 492)

PFNA = Proximal Femoral Nail Antirotation; TFN = Trochanteric Fixation Nail; TFNA = Trochanteric Fixation Nail Advanced.

### Association Between Patient Factors and Complications

Anticoagulation (OR 1.70 [95% CI 1.11 to 2.59]; p = 0.01), congestive heart failure (OR 1.91 [95% CI 1.13 to 3.11]; p = 0.01), and hypertension (OR 1.67 [95% CI 1.08 to 2.63]; p = 0.02) were associated with a major complication. Liver disease (OR 5.19 [95% CI 0.78 to 20.8]; p = 0.04) was associated with a major complication leading to reoperation (Table 4).

**Table 4.** Unadjusted associations of patient characteristics and major complications calculated using univariable logistic regression analyses, with complications set to the dependent variable and each patient characteristic as an independent variable

Variable	Any major complication		Complication leading to reoperation	
	OR (95% CI)	p value	OR (95% CI)	p value
Cardiac insufficiency	1.91 (1.13 to 3.11)	0.01	1.02 (0.41 to 2.16)	0.97
Myocardial infarction	1.05 (0.36 to 2.46)	0.91	0.82 (0.13 to 2.73)	0.78
PVD or CAD	1.45 (0.92 to 2.24)	0.10	1.15 (0.59 to 2.11)	0.67
Stroke	0.88 (0.47 to 1.54)	0.68	0.34 (0.08 to 0.93)	0.07
Hemiparesis	1.16 (0.34 to 2.96)	0.79	1.14 (0.18 to 3.85)	0.63
Dementia	0.77 (0.48 to 1.19)	0.25	0.76 (0.39 to 1.40)	0.40
Diabetes	1.33 (0.80 to 2.13)	0.26	1.22 (0.58 to 2.33)	0.58
Liver insufficiency	2.46 (0.37 to 9.71)	0.25	5.19 (0.78 to 20.8)	0.04
Connective tissue disease	1.03 (0.45 to 2.07)	0.94	1.32 (0.45 to 3.11)	0.57
COPD	0.59 (0.18 to 1.46)	0.32	0.29 (0.02 to 1.34)	0.22
Tumor	1.11 (0.61 to 1.90)	0.71	0.93 (0.38 to 1.97)	0.86
Renal insufficiency	1.94 (0.83 to 4.01)	0.10	0.41 (0.02 to 1.92)	0.38
Hypertension	1.67 (1.08 to 2.63)	0.02	1.58 (0.88 to 2.98)	0.14
Osteoporosis	0.80 (0.33 to 1.66)	0.58	0.93 (0.28 to 2.34)	0.89
Mental illness	1.50 (0.51 to 3.59)	0.41	1.14 (0.18 to 3.85)	0.86
Intoxification injury^a^	1.79 (0.72 to 3.86)	0.17	2.01 (0.59 to 5.20)	0.20
Smoking	0.98 (0.40 to 2.04)	0.56	0.88 (0.26 to 2.21)	0.81
Anticoagulation	1.70 (1.11 to 2.59)	0.01	1.10 (0.33 to 2.86)	0.82
Living at home	1.13 (0.72 to 1.82)	0.60	1.20 (0.65 to 2.38)	0.57
Age	1.00 (1.00 to 1.01)	0.81	1.00 (1.00 to 1.00)	0.91
Sex	1.09 (0.69 to 1.79)	0.71	1.49 (0.77 to 3.19)	0.27
Time to surgery	1.10 (0.90 to 1.34)	0.33	1.15 (0.87 to 1.49)	0.32

The results are presented as odds ratios along with 95% CIs. All complications and complications leading to reoperation were analyzed separately.

a Femur fracture while under the influence of alcohol. PVD = peripheral vascular diseases; CAD = coronary artery disease; COPD = chronic obstructive pulmonary disease.

## Discussion

New-generation nail types are comparable options based on the reoperation risk. Anticoagulation, congestive heart failure, and hypertension were associated with major complications in this study, highlighting the need for careful management and monitoring of these comorbidities during intramedullary nailing procedures.

### Limitations

First, because of the retrospective design of the study, we cannot be certain that all complications were diagnosed, especially in patients who died soon after having the operation. Although we assume our study provides valuable insight into the distribution of major complications, the reported risks should be seen as conservative estimates, providing a lower bound for the occurrence of complications.

Second, some patients with postoperative medical complications, such as pneumonia, can also be treated in primary healthcare settings without any contact with the hospital. Therefore, the proportion of patients experiencing these complications could be much higher than reported. However, we assume the complications that were defined as “major” are especially well detected because they require treatment in a hospital.

Third, in this study, urinary tract infection, delirium, and need for blood transfusion were not defined as complications. Including these complications would have affected the analysis and thus our conclusions.

Fourth, the tip-apex distance was not evaluated in our study, and we acknowledge this limitation prevents us from drawing definitive conclusions regarding the specific reasons for fixation failure. Further investigations that assess the tip-apex distance would be beneficial in providing a more comprehensive understanding of the outcomes associated with different nail types.

Finally, the study was not multicentered, and as a result, the results of this study may not be generalizable to lower-volume hospitals. However, the results of the study can be applied to other high-volume hospitals and teaching hospitals, which typically handle a large number of procedures and have experienced surgical teams and specialized facilities.

### Major Complications and 30-day Mortality

Our results provide a comprehensive overview of the occurrence of major complications, including surgical and medical complications, that may have a major impact on patients' well-being or may result in prolonged hospitalization or surgical procedures.

In earlier studies, the overall proportion of complications has varied widely, between 8.1% and 75% [1, 9, 27, 31] (Table 5). In the current study, urinary tract infection, delirium, and transfusions were not defined as major complications, which explains the lower proportion of patients experiencing complications in our study. In general, the 30-day mortality of patients with hip fractures varies between 5% and 10.5% [14, 24], which is in line with the findings of the present study. The risk of postoperative pneumonia has varied between 4.2% and 10% [1, 7, 9, 19, 27, 31]. Because some patients with postoperative pneumonia are treated in primary healthcare settings without contacting the hospital, the risk of pneumonia presented in this study could be higher than reported (2.7%). The risk of myocardial infarction has varied between 1.1% and 2.9% [5, 26, 27, 31]. These results are in line with our findings of a 2.5% incidence of myocardial ischemia. Targeting patients who have specific comorbidities, including cardiovascular diseases and diabetes, could reduce the risk of myocardial infarction and other cardiovascular complications [26]. A few studies reported a wound infection risk between 0.8% and 2.8% [2, 12, 27], which is similar to our results. Furthermore, another study described surgical delay as a risk factor for wound infection after hip fracture treatment [7]. Hence, early fixation (within 24 hours) may reduce the risk of surgical site infections. Prior studies found that cutout is the most frequent implant-related complication in patients with proximal femoral fractures, with a risk between 1% and 5% [25, 30, 33]. Similarly, in our study, the risk of cutout was 2.3%. It has been reported that achieving the appropriate tip-apex distance can reduce the risk of cutout, while a tip-apex distance greater than 25 mm may increase the risk of cutout [29]. In the present study, there was one perioperative iatrogenic femoral fracture. However, there were no postoperative peri-implant fractures around the distal tip of the nail. A systematic review of 13,568 patients reported that the incidence of postoperative peri-implant fractures varied between 0% and 2.3%, suggesting that continuing changes in the design of intramedullary nails of the proximal femur have reduced the risk of this complication [23].

**Table 5.** Comparison table of clinical studies that investigated complications after surgery for hip fractures

Author	Year	Type and design	Patients	Overall proportion of patients experiencing complications	Surgical complications	Medical complications
Belmont et al. [1]	2014	National database study	9286	12.5%	Wound infections: 1.4%	Pneumonia: 4.6%
Bojan et al. [2]	2010	Retrospective single-center study	3066	Medical and surgical complications analyzed separately	Intraoperatively: 4.5%	Overall: 5.6%
					Postoperatively: 6.2%	
Flikweert et al. [9]	2017	Prospective cohort study	479	75%	Wound problems: 9%	Pneumonia: 10%
					Implant-related: 4%	
Remily et al. [27]	2020	National database study	2,761,850	Varied between 26.8% and 29.5% (when transfusions were excluded)	Mechanical complications: 0.9% to 1.1%	Myocardial infarction: 1.7% to 2.4%
						Pulmonary emboli: 0.7% to 0.8%
					Hematoma or seromas: 0.8 to 1.3%	Pneumonia: 4.2% to 5.5%
						Sepsis: 1.8% to 2.6%
Sheehan et al. [31]	2017	National database study	153,613	8.1%	Included only medical complications	Pneumonia: 4.9%
						Shock or myocardial infarction: 1.1%
						Sepsis: 0.4%
						Deep venous thrombosis or pulmonary embolism: 1.1%

This table focuses only on the complications that were collected and analyzed in our study. Other complications that were not part of our data collection are not included in this table.

### Association Between Implant Types and Complications

Our results support that the new-generation nail types are comparable options to treat intertrochanteric fractures; we found that nail type was not associated with the odds of major complications leading to reoperation. A retrospective study of 7979 patients compared the revision risk between the TFNA and TFN, and after adjustment for covariates, there was no difference in the risk of revision [11], which is consistent with our results. In the present study, the risk of cutout was 3.0% for Gamma3 and 2.0% for TFNA, whereas another study [30] reported a 2.2% risk of cutout for Gamma3 and 4.8% for TFNA. However, the risks of cutout were similar to those of our results when comparing femoral neck screws and helical blades, because we found that 2.1% of femoral neck screws and 3.3% of helical blades cut out. In that previous study, a helical blade was used in 74% of patients in the TFNA group [30], whereas Gamma3 nails incorporated a femoral neck screw. In two clinical studies reporting similar results, the cutout risk was higher when a helical blade was used than when a femoral neck screw was used for fixation [4, 32]. Another study highlighted higher cutout rates with Gamma3 than with the Trochanteric Gamman Nail, suggesting that a lag screw, which has a smaller diameter in the G3 design than in the Trochanteric Gamman Nail design, may decrease the resistance of migration to the femoral head [21]. However, the proper position of the blade or screw may be the key to minimize cutout rather than the design of the implant [22, 34]. Although nail breakages are reported infrequently, an implant retrieval study of 16 broken TFNA nails reported a unique fracture pattern with a stepped propagation pathway [16]. A retrospective study of 7979 patients [11] comparing the revision risk between the TFNA and TFN reported implant breakage of 0.06% for the TFN and 0.2% for TFNA. Furthermore, in two recent studies, the risk of nail breakage was similar between the TFNA and comparator intramedullary nails [30, 35]. In our study, the risk of nail breakage was 0.8% among patients treated with TFNA, whereas it was 0.7% for those with PFNA and 0.3% for those with Gamma3. Hence, our study, along with recent research, suggests that nail breakages are rare events across different nail types.

### Association Between Patient Factors and Complications

In the present study, congestive heart failure, hypertension, and anticoagulation each showed an association with a major complication. This highlights the need for careful management and monitoring of these comorbidities during intramedullary nailing procedures. Similarly, one study [1] reported cardiac disease was a risk factor for a major complication, and hypertension was identified as being related to an increased odds of pulmonary complications after hip fracture. Studies investigating the postoperative outcomes of patients with anticoagulated hip fractures have been mixed [13, 17]. Patients using anticoagulation often experience delays in surgical fixation, which can increase their risk of complications [17]. However, a cross-sectional analysis [13] found no difference in outcomes between patients using anticoagulants and those not using them, after adjusting for patient characteristics. In our study, delay of surgery was not associated with major complication. This was perhaps because patients generally underwent surgery on the day after fracture, so the operation was postponed for longer than 2 days for only a small proportion of patients (14%). In our study, liver disease was associated with a major complication leading to reoperation. However, the value of 1 was included in the 95% CI, indicating that the association cannot be demonstrated at a 95% confidence level, even though the p value is below 0.05. Hence, it is not possible to draw definitive conclusions with the limited sample size of only 11 patients with liver failure, which is insufficient for adequate parametric statistical testing.

### Conclusion

This study provides a better understanding of the occurence of surgical and medical complications after intramedullary nailing of intertrochanteric fractures. The new-generation nail types are comparable options based on the reoperation risk. Anticoagulation, congestive heart failure, and hypertension were associated with major complications, highlighting the need for careful management and monitoring of these comorbidities during intramedullary nailing procedures.
